# Pulmonary embolism during pregnancy: a 17-year single-center retrospective MDCT pulmonary angiography study

**DOI:** 10.1007/s00330-019-06501-4

**Published:** 2019-11-14

**Authors:** David C. Rotzinger, Vincent Dunet, Vesna Ilic, Olivier W. Hugli, Reto A. Meuli, Sabine Schmidt

**Affiliations:** 1 Department of Diagnostic and Interventional Radiology, Emergency Radiology Division, Lausanne University Hospital (CHUV), Rue du Bugnon 46, 1011 Lausanne, Switzerland; 2 Faculty of Biology and Medicine (FBM), University of Lausanne (UNIL), Lausanne, Switzerland; 3 Centre d’Imagerie du Nord Vaudois (CINOV), Yverdon-les-Bains, Switzerland; 4 Emergency Department, Lausanne University Hospital (CHUV), Lausanne, Switzerland

**Keywords:** Multidetector computed tomography, Computed tomography angiography, Pulmonary embolism, Hematologic pregnancy complications

## Abstract

**Objectives:**

To determine the prevalence of pulmonary embolism (PE) and alternative diagnoses detected by computed tomography pulmonary angiography (CTPA) in pregnant women; and to assess changes over time regarding radiation dose, technical quality, and examination frequency.

**Materials and methods:**

This retrospective study included all pregnant women referred for CTPA due to clinically suspected PE over 17 years. Two blinded radiologists reviewed the CTPAs in consensus with regard to PE, alternative diagnoses, and technical quality. We retrieved patient data regarding radiation dose metrics and associated clinical and laboratory parameters. Subgroup comparisons were performed (Wilcoxon and Kruskal-Wallis tests).

**Results:**

Of the 237 identified patients, 8 (3.3%) were excluded due to inadequate technical CTPA quality, and 229 patients were analyzed (mean age, 31.7 years; mean gestational age, 28 ± 7 weeks). The four different CT systems used over the study period had similar technical quality (*p =* 0.28). Of 229 patients 16 (7%) patients had PE, 144 (62.9%) had no abnormal findings, and 69 (30.1%) had an alternative diagnosis (consolidation, other pulmonary opacities, pleural effusion, and basal atelectasis). Gestational age, symptoms, and D-dimer levels were not significantly different between patients with or without PE (*p >* 0.05). Over time, radiation dose exposure decreased by 30% (*p <* 0.001), while the number of annual examinations increased by > 4-folds.

**Conclusions:**

In pregnant women, CTPA rarely indicates PE and more often shows alternative diagnoses. Over 17 years, the use of CTPA in pregnancy has notably increased, while the radiation dose exposure has decreased by one third.

**Key Points:**

*• The use of CTPA in pregnancy has steadily risen over the last 17 years*

*• In pregnant women, CTPA rarely reveals PE and more often shows alternative diagnoses*

*• Recent technical improvements have substantially decreased the radiation dose exposure inherent in CTPA without reducing diagnostic image quality*

## Introduction

Pregnancy induces a prothrombotic state, with increased coagulation factors, decreased natural anticoagulants, and impairment of fibrinolysis. This hypercoagulable state most likely evolved during pregnancy to protect women from the risk of bleeding during miscarriage and childbirth. Several factors can increase the risk for venous thromboembolism (VTE) during pregnancy, including inherited thrombophilia, antiphospholipid syndrome, or previous history of thrombosis [1–3]. Compared to the non-pregnant population, pregnancy increases the risk of deep vein thrombosis (DVP) and pulmonary embolism (PE) by at least four times. Acute VTE events occur in 1–2 per 1000 pregnancies [3, 4]. PE is the leading non-obstetric cause of maternal death, with death occurring in 1 of 100 pregnant women diagnosed with PE [4].

Typical PE symptoms are non-specific during pregnancy, since normal pregnancies commonly involve dyspnea, tachycardia, and leg swelling [5]. Likewise, clinical probability scores, such as the Wells model [6] and Geneva criteria [7], have not been validated in pregnant women [8]. Moreover, the progression of a normal pregnancy involves a physiological increase of D-dimer levels and current evidence suggests that a D-dimer test—even using pregnancy-specific thresholds—should not be used as a stand-alone test to manage pregnant patients with suspected PE [9].

Due to these diagnostic difficulties, pregnant women with suspected PE require additional diagnostic imaging, particularly if compression ultrasound (CUS) of the legs is negative or not indicated due to the absence of leg symptoms. Despite well-established guidelines [10] recommending chest X-ray followed by perfusion scintigraphy, computed tomography pulmonary angiography (CTPA) is preferred over ventilation-perfusion lung scan (V/Q) in most North American [11] and European centers. CTPA has the advantage of being available around the clock, potentially unveiling alternative diagnoses [4, 12, 13]. Moreover, CTPA can accurately exclude clinically significant PE in pregnancy [14]. Even when using a low-dose protocol, CTPA is associated with radiation exposure to the mother and the fetus; however, this is considered warranted given the critical nature of PE [15].

In the present study, we aimed to assess the prevalence of CTPA-detected PE in a population of pregnant women, as well as the prevalence of associated or alternative diagnoses, and the clinical factors associated with PE. Secondary objectives included the assessment of qualitative image quality and dose exposure over a long period.

## Materials and methods

This study was approved by our institutional ethics committee, and the requirement of informed consent was waived.

### Study design and patients

In this single-center tertiary care retrospective study, we performed a systematic query of our computerized institutional database to identify all of the radiological reports from patients referred to our emergency department due to clinically suspected acute PE. A search using the four keywords “pulmonary embolism,” “pregnancy,” “pregnant,” and “computed tomography” yielded records of 237 CTPAs performed in pregnant patients from January 2000 to August 2018.

### MDCT protocols

Over the study period, CTPA was performed using four different CT systems: a 4-row multidetector computed tomography (MDCT) system (LightSpeed QX/I; GE Healthcare) was used from January 2000 to November 2002 (14 examinations). A 16-row MDCT system (LightSpeed Ultra; GE Healthcare) was used from December 2002 to January 2006 (20 examinations). A 64-row MDCT system (LightSpeed VCT; GE Healthcare) was used from February 2006 to August 2015 (140 examinations). Finally, a 256-row MDCT system (Revolution CT; GE Healthcare) was used from September 2015 to August 2018 (63 examinations). Table 1 presents the detailed acquisition parameters for each of the four MDCT systems. All examinations were performed in helical acquisition mode. A power injector was used for intravenous (IV) injection of 80–120 mL iohexol (300 mg I/mL, Accupaque® 300; GE Healthcare) into an antecubital vein at a flow rate of 4–5 mL/s. Bolus tracking (SmartPrep) was applied, with the region of interest (ROI) centered on the main pulmonary artery. Acquisition was triggered when the CT attenuation exceeded 150 Hounsfield units. Patients were in a supine position, with their arms above their head, and were scanned from the diaphragm to the lung apices, at full inspiration. Starting in December 2016 (when using the 256-row MDCT system), we used three different tube potential settings (80, 100, or 120 kVp) depending on the patient’s body mass index (BMI): < 24, 24–26, or > 26 kg/m^2^, respectively. The image reconstruction parameters were as follows: section thickness, 2.5 mm until January 2006 and 1.25 mm from February 2006; section overlap, 2.25 mm until January 2006 and 1 mm from February 2006; kernel, soft tissue; algorithm, FBP; display field-of-view, adjusted to the patient’s size. We retrieved the volume CT dose indexes (CTDI_vol_) and dose-length products (DLP) from the dose exposure reports integrated into the DICOM images for each MDCT examination.

**Table 1:** Acquisition parameters of the four different MDCT systems

Parameter	MDCT system			
Detector configuration	4-row	16-row	64-row	256-row
Tube potential, kVp	120	120	120	80, 100, or 120*
Noise index	N/A	18	18	15.5
Tube current, mA	170	180	100–300	100–580
Beam collimation, mm	4 × 3.75	16 × 1.25	64 × 0.625	128 × 0.625
Beam pitch	1.2	1.75	0.984	0.992
Gantry rotation time, s	0.8	0.5	0.6	0.5
Acquisition direction	Caudocranial	Caudocranial	Caudocranial	Craniocaudal
Number of examinations per year	4.8	6.3	14.6	21.0

*MDCT* multidetector computed tomography

*Depending on patient body mass index (BMI)

### Image analysis

All MDCT images were analyzed using a picture archiving and communication system workstation (Vue PACS version 11.4; Carestream Health) and displayed with the default soft tissue window (level, 50 HU; width, 350 HU). Raters were able to change windowing as needed to optimize vessel visualization. Each CTPA examination was reviewed by two radiologists (S.S. and V.I.) with 14 and 4 years of experience in thoracic imaging, respectively, who were blinded to all clinical and radiological information. Images were reviewed in consensus and not separately, since the interrater agreement for CTPA evaluation has already been assessed before [13, 14, 16].

On a per-examination basis, we visually evaluated the enhancement quality of the pulmonary arteries by the IV-injected contrast agent using a 4-point Likert scale: 0 = non-diagnostic (excluded), 1 = diagnostic image with presence of severe artifacts, 2 = good image quality with minor artifacts, and 3 = excellent image quality with no relevant artifacts [17]. The examinations were then classified as positive or negative for acute PE. Signs of acute PE were defined as a luminal filling defect that was either totally occlusive in a normal-sized or enlarged vessel, or centrally located and delineated by contrast medium [18]. We recorded the anatomical location of acute PE (i.e., main, lobar, segmental, and sub-segmental), and measured the maximal diameter of the pulmonary trunk. We also noted the following diagnoses—pulmonary consolidation, any pulmonary opacities other than consolidation (tree-in-bud pattern or ground-glass opacity), basal band-like atelectasis, and pleural effusion—that could occur in association with PE or as an alternative diagnosis in the absence of PE.

After evaluating the CTPA images, we searched the patients’ clinical charts using a structured approach to retrieve the patients’ characteristics, history, symptoms, blood test values, and risk factors for VTE (i.e., immobilization, obesity, thrombophilia, previous VTE, and smoking). From the patients’ past medical history, we retrieved data regarding underlying heart disease, arterial hypertension, multiple gestations, parity, and use of assisted reproductive techniques. Presenting symptoms included sudden onset of chest pain, dyspnea, hemoptysis, desaturation (oxygen saturation of < 90% by pulse oximetry), or uni- or bilateral leg edema. We also recorded the following additional clinical, laboratory, and anamnestic parameters: tachycardia (heart rate > 110 beats/min), S1Q3T3 pattern on ECG, D-dimer levels, CUS of the legs before CTPA, and ongoing therapeutic anticoagulation.

### Statistical analysis

All statistical analyses were performed using Stata 13.1 software (StataCorp). Continuous variables are presented as mean ± standard deviation (SD) and categorical variables as numbers or proportions. The proportions of patients with PE, an alternative diagnosis, and no radiological findings were defined as the percentages of patients in these three categories among the whole study cohort. Between-group data comparisons were performed in a two-step fashion. Firstly, we compared patients with PE to patients without PE. Secondly, we made comparisons between patients with PE, patients with an alternative diagnosis and those with a normal CTPA (i.e., without any radiological findings). Between-group comparisons were performed using the Wilcoxon sign rank-sum or Kruskal-Wallis tests for continuous variables, and Fisher’s exact test or the Chi-squared test, as appropriate, for categorical variables. For variables that significantly differed between groups, we performed logistic regression analysis with computation of the odds ratio (OR) and 95% confidence interval (CI). The Kruskal-Wallis test was used to compare image quality and dose parameters between the four MDCT scanners. A *p* value of < 0.05 was considered statistically significant. When needed, the significance level was corrected for multiple comparisons using the Bonferroni method.

## Results

### Patients and diagnoses

**Fig. 1:**
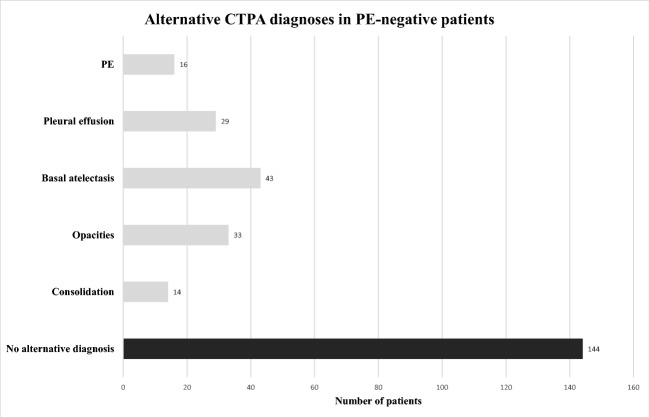
Histogram shows the computed tomography pulmonary angiography (CTPA) findings in the patients without pulmonary embolism (PE) (*n* = 213), including alternative diagnoses, and completely normal CT examinations (*n* = 144, 67.6%)

**Fig. 2:**
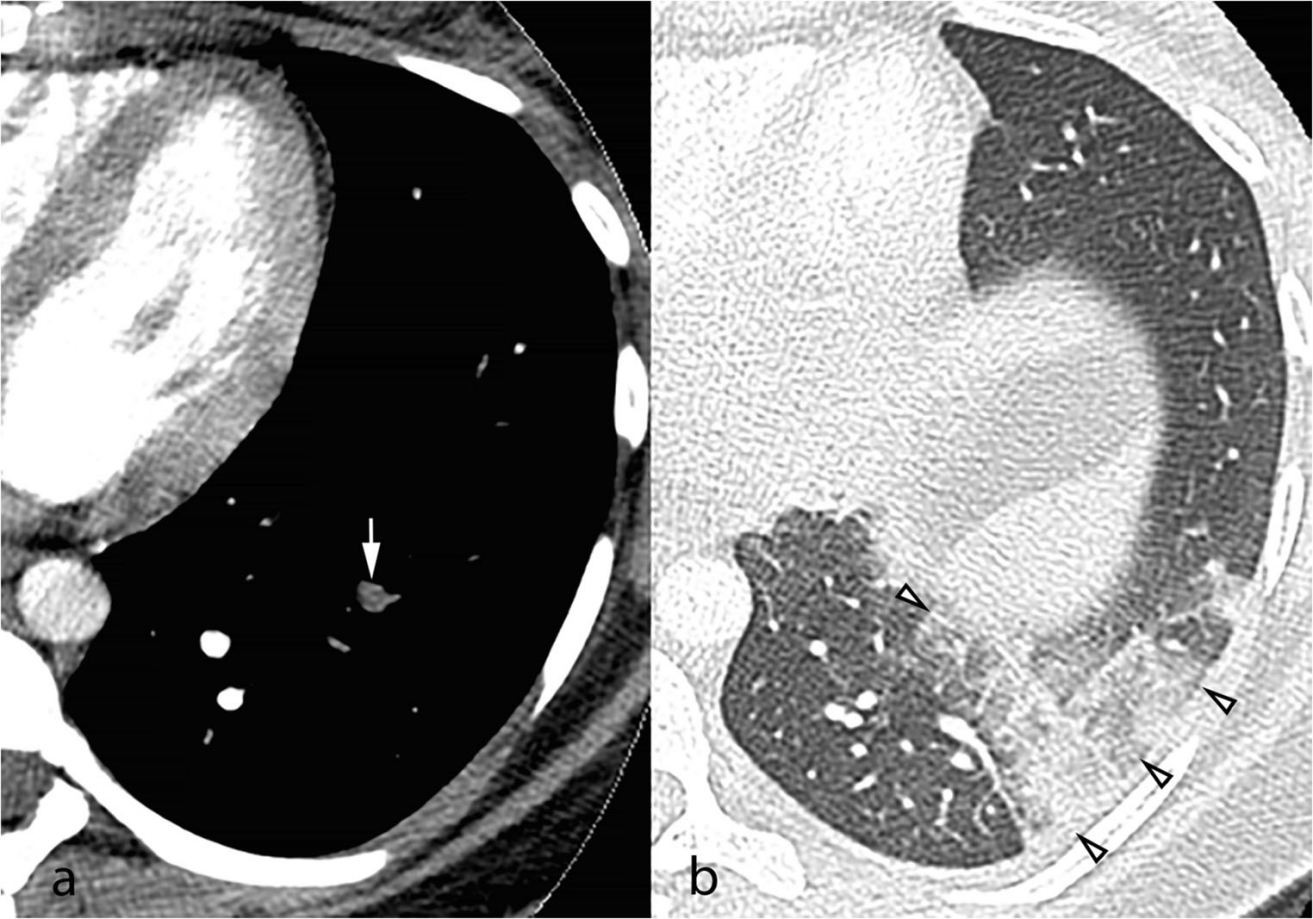
Axial computed tomography pulmonary angiography images in the soft tissue (**a**) and lung windows (**b**), from a 25-year-old pregnant patient presenting with acute chest pain. A segmental pulmonary embolus was detected in the left lower lobe (**a**, white arrow), with associated triangular-shaped areas of subpleural alveolar density (**b**, arrowheads), consistent with pulmonary infarction

**Fig. 3:**
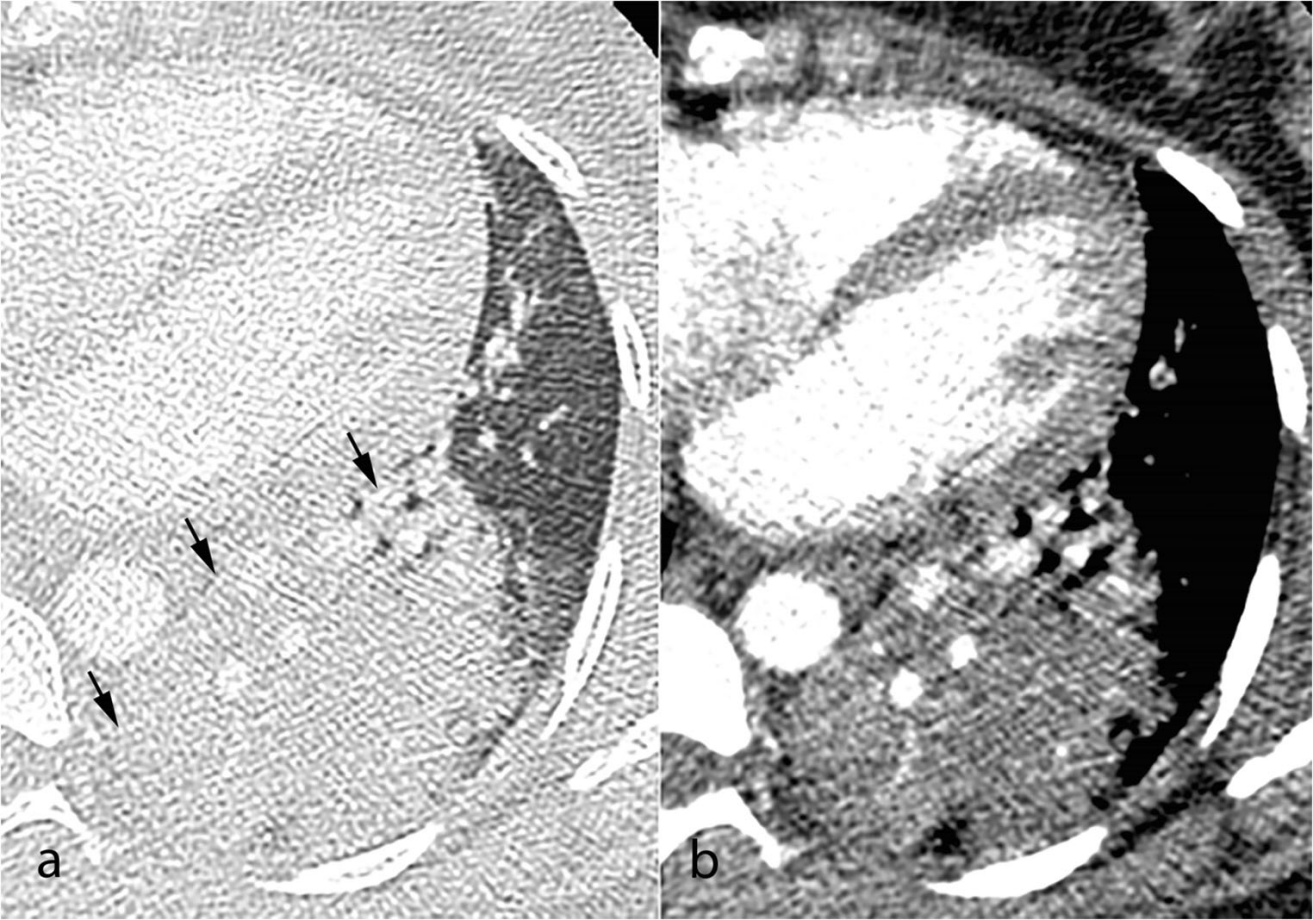
Axial computed tomography pulmonary angiography images in the lung (**a**) and the soft tissue windows (**b**), from a 33-year-old pregnant patient presenting with acute chest pain and dyspnea. No pulmonary emboli were detected; however, the scan revealed left lower lobe consolidation consistent with pulmonary infection (**a**, black arrows)

Of the 237 identified patients, 8 (3.3%) were excluded from analysis due to poor technical image quality*.* In 7 patients (88%), the pulmonary arteries were too poorly enhanced and in one patient, whose CTPA revealed left lower lobe pneumonia, there were too many respiratory artifacts. None of these 8 excluded patients underwent further diagnostic imaging, and they were thus considered negative for VTE. Our analysis included a total of 229 patients with a mean age of 31.7 ± 5.7 years (range 18–49 years), and a mean gestational age of 28 ± 7 weeks (range 6–40 weeks). Among these patients, 16 (7%) had acute PE, 69 (30.1%) had an alternative diagnosis, and 144 (62.9%) exhibited no abnormal findings upon CTPA (Fig. 1). Clinically, pulmonary infection with/without pleural effusion was diagnosed in 17 patients (24.6%), fluid overload with pulmonary opacities (mostly of cardiac, renal, or septic origin) with/without pleural effusion in 21 patients (30.4%), basal atelectasis (mostly due to immobilization or abdominal diseases) with/without pleural effusion in 21 patients (30.4%), pulmonary opacities of another origin (i.e., interstitial lung disease, ARDS) in 8 patients (11.6%), and pleural effusion only in 2 patients (3%). Figures 2 and 3 provide examples of acute PE with associated pulmonary infarction and pulmonary consolidation, thus pulmonary infection, in pregnant patients.

In patients with PE, the position of the most proximal clot was identified as proximal (*n* = 1), lobar (*n* = 4), segmental (*n* = 10), or sub-segmental (*n* = 1). Of the 16 patients with PE, 8 had associated findings, including pulmonary opacities (*n* = 2), pleural effusion (*n* = 2), or basal atelectasis (*n* = 4). Among the 213 patients without PE, 35 (16.4%) had a single alternative diagnosis, while 34 (16.0%) had multiple alternative diagnoses. Mean pulmonary trunk diameter was 26 ± 2.8 mm among PE-positive patients, and 27 ± 3.7 mm among PE-negative patients (*p* = 0.97). Of the 229 patients included in the analysis, 16 (7%) underwent a chest X-ray before CTPA, and 88 (38.4%) underwent a lower extremity CUS that yielded negative results before CTPA. Among the 16 patients with PE, 7 (43.8%) had a normal CUS of the lower extremities, and 3 (18.8%) underwent a chest X-ray before CTPA. After CTPA, perfusion scanning was performed in 8 (3.3%) of the 229 patients, but not in any patient in whom the CTPA findings indicated PE. These perfusion scans were negative for PE except in one case where the result was inconclusive because of minor bilateral subpleural perfusion abnormalities. In one patient (0.5%), CTPA was performed following an inconclusive perfusion scan.

### Clinical characteristics associated with PE

**Fig. 4:**
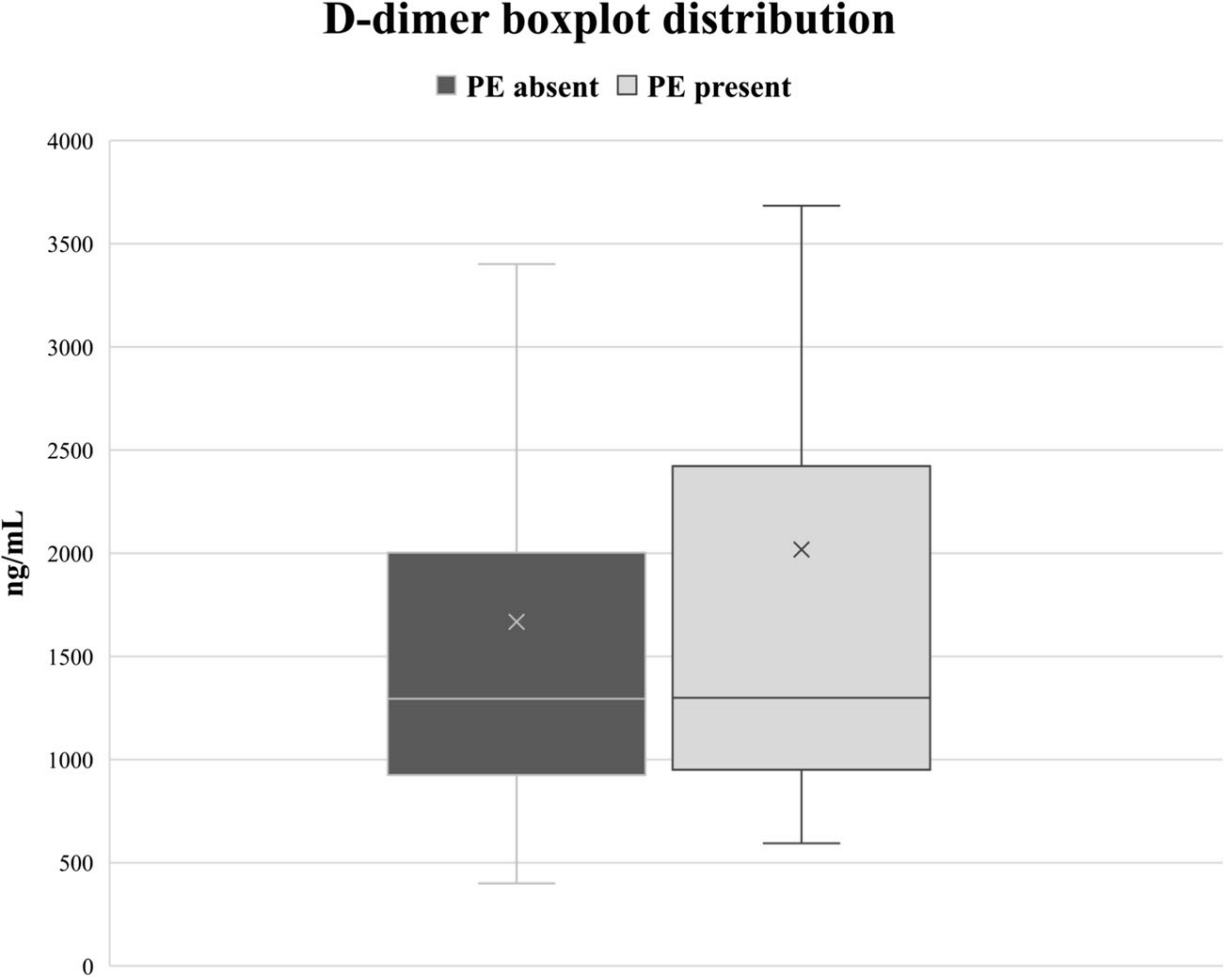
D-dimer boxplot distribution in patients with and without pulmonary embolism (PE) detected on computed tomography pulmonary angiography

Table 2 presents the baseline characteristics, risk factors, medical history, symptoms, presence of ECG abnormalities, and D-dimer levels for PE-positive and PE-negative patients, with univariate comparison. None of the evaluated parameters significantly differed between the two groups. Chest pain was present in 93.8% of patients with PE, and 72.3% of patients without PE, but this difference was not statistically significant (*p* = 0.076). D-dimer levels did not significantly differ between the two groups (*p* = 0.91), as illustrated in Fig. 4.

**Table 2:** Characteristics, risk factors, anamnestic parameters, and symptoms—compared between PE-positive and PE-negative patients by univariate analysis

Variables	PE positive (*n* = 16)	PE negative (*n* = 213)	*p* value
*Characteristics*			
Age, years	29.8 ± 5.6	31.9 ± 5.7	0.17
Gestational age, weeks	27.3 ± 8.1	27.8 ± 7.7	0.81
Anticoagulation	0 (0.0%)	15 (7.0%)	0.61
*Risk factors*			
Immobilization	2 (12.5%)	40 (1.9%)	0.74
Obesity	1 (6.3%)	24 (11.3%)	1.0
Thrombophilia	0 (0.0%)	10 (4.7%)	1.0
Previous VTE	0 (0.0%)	9 (4.25)	1.0
Smoking	1 (6.3%)	20 (9.4%)	1.0
*History*			
Heart disease	0 (0.0%)	9 (4.2%)	1.0
Hypertension	0 (0.0%)	11 (5.2%)	1.0
Multiple gestations	2 (12.5%)	26 (12.2%)	1.0
Parity	0.94 ± 1.18	0.66 ± 1.05	0.27
Assisted reproductive techniques	0 (0.0%)	5 (2.3%)	1.0
*Symptoms*			
Chest pain	15 (93.8%)	154 (72.3%)	0.076^#^
Dyspnea	12 (75.0%)	167 (78.4)	0.76
Hemoptysis	2 (12.5%)	6 (2.8%)	0.10
Desaturation (*O*_*2*_*saturation <* 90%)	6 (37.5%)	43 (20.2%)	0.12
Lower extremity swelling	5 (31.3%)	31 (14.6%)	0.14
*Other parameters*			
Tachycardia *	5 (31.3%)	48 (23.8%)	0.51
Abnormal ECG	1 (6.3%)	21/213	1.0
D-dimers, ng/mL †	2238 ± 2056	1552 ± 1241	0.91

*PE* pulmonary embolism, *VTE* venous thromboembolism, *ECG* electrocardiogram

†D-dimers were available in 176 (78.2%) patients, including 8 with PE and 168 without

*Heart rate information was available for 218 (95.2%) patients, including 16 with PE and 202 without

^#^Patients with PE tended to more commonly experience chest pain

The cohort was subdivided into three groups: PE; alternative diagnosis; and no radiological diagnosis (Table 3). Subgroup analysis showed that desaturation was more frequent in patients with positive CTPA findings, being noted in 37.5% of PE patients, and 33.3% of patients with an alternative diagnosis, compared to only 13.4% of patients with no radiological diagnosis (*p* = 0.001). Likewise, uni- or bilateral leg edema was significantly more frequent in PE patients (31.3%), than in patients with an alternative diagnosis (8.7%) or no diagnosis (17.4%) (*p* = 0.045).

**Table 3:** Characteristics, risk factors, anamnestic parameters, and symptoms—compared among the three patient groups

Variables	PE positive (*n* = 16)	Alternative diagnosis (*n* = 69)	No findings on CTPA (*n* = 144)	*p* value
*Characteristics*				
Age, years	29.8 ± 5.6	32.2 ± 5.6	31.7 ± 5.9	0.27
Gestationnal age, weeks	27.3 ± 8.1	27.8 ± 7.3	27.9 ± 7.9	0.83
Anticoagulation	0 (0%)	2 (2.9%)	13 (9.0%)	0.18
*Risk factors*				
Immobilization	2 (12.5%)	18 (26.1%)	22 (15.3%)	0.17
Obesity (BMI > 30 kg/m^2^)	1 (6.3%)	5 (7.2%)	19 (13.2%)	0.46
Thrombophilia	0 (0.0%)	1 (1.5%)	9 (6.3%)	0.29
Previous VTE	0 (0.0%)	1 (1.5%)	8 (5.6%)	0.43
Smoking	1 (6.3%)	8 (11.6%)	12 (8.3%)	0.75
*History*				
Heart disease	0 (0.0%)	5 (7.2%)	4 (2.8%)	0.24
Hypertension	0 (0.0%)	6 (8.7%)	5 (3.5%)	0.21
Multiple gestations	2 (12.5%)	11 (15.9%)	15 (10.4%)	0.48
Parity	0.94 ± 1.18	0.64 ± 1.11	0.67 ± 1.02	0.38
Assisted reproductive techniques	0 (0.0%)	3 (4.3%)	2 (1.4%)	0.54
*Symptoms*				
Chest pain	15 (93.8%)	52 (75.4%)	102 (70.8%)	0.13
Dyspnea	12 (75.0%)	57 (82.6%)	110 (76.4%)	0.55
Hemoptysis	2 (12.5%)	2 (2.9%)	4 (2.8%)	0.17
Desaturation (*O*_*2*_*saturation <* 90%)	6‡ (37.5%)	23§ (33.3%)	20 (13.8%)	*0.001*
Lower extremity swelling	5° (31.3%)	6 (8.7%)	25 (17.4%)	*0.045*
*Other parameters*				
Tachycardia*	5 (31.3%)	22 (34.4%)	26 (19%)	0.056
S1Q3 EKG	1 (6.3%)	5 (7.2%)	16 (11.1%)	0.71
D-dimers, ng/mL†	2238 ± 2056	1550 ± 824	1553 ± 1377	0.71

Italicized *p* values are statistically significant

*PE* pulmonary embolism, *CTPA* computed tomography pulmonary angiography, *VTE* venous thromboembolism, *ECG* electrocardiogram

†D-dimer levels were available for 176 patients (78.2%), including 8 with PE, 48 with an alternative diagnosis and 120 with no radiological findings

*Heart rate information was available in 218 patients (95.2%), including 16 with PE, 64 with an alternative diagnosis and 137 with no radiological findings

§*p* = 0.001 compared to patients without radiological diagnosis

‡*p* = 0.016 compared to patients without radiological diagnosis

°*p* = 0.029 compared to patients with an alternative diagnosis

Logistic regression analysis revealed that desaturation predicted an abnormal CTPA (PE or an alternative diagnosis) compared to no radiological findings (OR 3.1, 95% CI 1.6–6.2, *p* < 0.001). Swelling of the legs predicted PE compared to an alternative diagnosis (OR 4.8, 95% CI 1.2–18.4, *p* = 0.023).

### Image quality and dose exposure

The visually assessed technical quality of each CTPA (i.e., the contrast medium enhancement of the pulmonary arteries) exhibited a similar distribution among the four CT types (*p* = 0.28). The mean scores were as follows: 4-MDCT, 2.1 ± 0.7 (*n* = 11); 16-MDCT, 2.6 ± 0.6 (*n* = 20); 64-MDCT, 2.5 ± 0.7 (*n* = 136); and 256-MDCT, 2.5 ± 0.7 (*n* = 62).

**Fig. 5:**
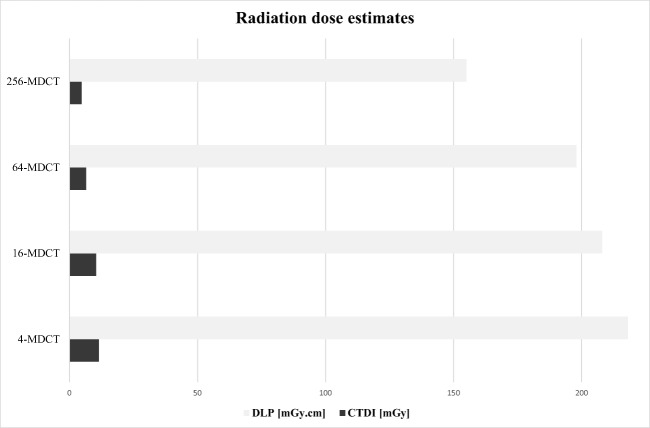
Histogram shows dose exposure estimates expressed as dose-length product (DLP) and volume computed tomography dose index (CTDI_vol_). MDCT multidetector computed tomography

Both CTDI_vol_ and DLP significantly differed between the four different MDCT systems (*p* < 0.001), showing a significant decrease with newer systems (Fig. 5). On the other hand, the number of CT examinations performed annually showed a gradual and substantial increase, ranging from 4.8 scans per year with the 4-row CT system, to 21 scans per year with the 256-row system, constituting a > 4-fold rise over the last 17 years (Table 1).

## Discussion

Our current results including 229 pregnant women indicate that 7% had acute PE, 30% had alternative diagnoses, and 63% had a normal CTPA. This low prevalence of PE indicates that CTPA was mainly useful for excluding PE in this population. Notably, alternative, and sometimes clinically important diagnoses were made from CTPA in one third of patients.

This was the first study to evaluate the use of CTPA for suspected PE in pregnant women over a period of 17 years. Over this timeframe, we observed a dramatic increase in the number of scans performed, starting from 4.8 examinations per year in 2000, and reaching 21 examinations per year by 2018, mirroring the results from an extensive single-center review performed in North America [19]. In that cohort, the rate of X-ray imaging in pregnant patients increased by > 100% over 10 years, mostly due to the use of CT. In our cohort, however, the rate of PE-positive CTPAs did not simultaneously increase over time, being 11.4% from January 2000 to November 2002, 0% from December 2002 to January 2006, 6.8% from February 2006 to August 2015, and 11.1%. from September 2015 to August 2018. Thanks to technological improvements across the study period, the total radiation dose (DLP) decreased by 30% per scan, and CTDI_vol_ dropped by almost 60%, from 11.5 to 4.8 mGy, without cutting back on image quality.

Overall, PE was diagnosed in 7% of patients, which is in agreement with previously reported data [20–24]. This rate of PE-positive CTPAs is about half the rate usually reported in the general non-pregnant population, confirming the challenging nature of clinical assessment for PE during pregnancy [25, 26]. This difficulty is highlighted by the absence of clinical symptoms showing significant predictive value for PE. Furthermore, no clinical or laboratory factor (Table 2) was found to predict PE presence on CTPA. This is in agreement with Touhami et al, who recently reported that the revised Geneva and Wells scores are not helpful for PE prediction [8]. Only after subdividing the cohort into 3 groups (PE positive, alternative diagnosis, no findings on CTPA, Table 3), desaturation appeared to be a predictor of an abnormal CTPA (PE or alternative diagnosis), and leg swelling appeared to be a predictor of PE compared to an alternative diagnosis. Both are known signs that can be found in PE-patients but offer little advantage in the diagnostic workup of pregnant patients. Based on the latest prospective studies, there is currently no consensus regarding the exact D-dimer threshold that should be applied during pregnancy. Righini et al [23] used a conservative threshold of 500 μg/L. Van der Pol et al [22] used 1000 μg/L for patients not meeting any YEARS criteria (clinical signs of DVT, hemoptysis, and PE being the most likely diagnosis), or 500 μg/L in the presence of one or more YEARS criteria. Our results showed equal elevation of D-dimer levels in women with and without PE, and D-dimer levels of < 1000 μg/L in one third of our patients with PE. These findings raise questions regarding the reliability of higher PE-specific D-dimer thresholds, as proposed by some authors [22].

In many hospitals worldwide and in our hospital, CTPA has become the first-line modality to exclude acute PE in pregnant women, despite the current guidelines advocating V/Q scanning, when available [27]. V/Q scanning following a normal chest X-ray remains a valid approach but is today often used as a second-line test, when CTPA is inconclusive or contraindicated, such as in patients with renal impairment or intolerance to iodinated contrast agents. Only 9 (3.9%) patients in our cohort underwent V/Q scanning. There are multiple reasons for the predominance of CTPA, such as the immediate and widespread availability, the fast acquisition time, the high accuracy with direct visualization of vascular emboli, and the ability to provide alternative diagnoses [28]. This latter point is critical and well reflected in our present study cohort, where CTPA revealed alternative diagnoses four times more often than the presence of PE. In a multicenter study including 512 pregnant women who underwent CTPA, van Strijen et al found alternative diagnoses in 25% of patients, similar to our study, consisting mostly of pneumonia [29]. In these patients, a chest X-ray, which is usually performed before V/Q scanning, would have been abnormal and, thus, casting doubt on the added value of V/Q scanning. Indeed, according to the most recent European guidelines, V/Q scanning is generally not performed in such patients [30]. Radiation risk is a continuing concern, especially in the pregnant population given the radiation sensitivity of both the fetus and the maternal breast. Since CTPA delivers slightly lesser dose to the fetus than V/Q scanning, but a substantially higher dose to the maternal breast tissue [31, 32], V/Q scanning following a normal chest X-ray is still recommended by many scientific societies [27, 33]. Either way, women subjected to ionizing radiation should be made aware that V/Q carries a slightly higher risk of childhood cancer than CTPA (1 in 280,000 vs. 1 in 1,000,000, respectively), whereas CTPA is associated with a slightly increased lifetime risk of breast cancer (increasing from 1 in 200 to 1.1 in 200) [34]. Even so, the radiation risks of both V/Q scanning and CTPA are well below the threshold for fetal complications and should not prevent pregnant women from receiving either imaging test [35, 36].

Finally, according to current guidelines [10], CUS of the lower extremities was performed only in patients showing signs and symptoms of DVT (i.e., leg pain or swelling), which was the case in 38.4% of patients. The remaining patients directly underwent CTPA.

Recently proposed management algorithms for acute PE suspicion in pregnant women do no longer include chest X-rays and focus on structuring the clinical and paraclinical workup to appropriately select patients who will likely benefit from CTPA [22, 23]. At our hospital, chest X-rays were avoided in most patients (93%) to reduce radiation exposure, based on the limited role of X-rays to drive management strategies in pregnant women [9]. Among the 16 PE-positive patients, 8 (50%) had parenchymal abnormalities on CTPA, and 2 (12.5%) had pleural effusion. This substantiates the potential diagnostic confusion that could be caused by chest X-ray in these pregnant women, as described by Goodacre et al [9].

Our study had several limitations. First, its retrospective nature entailed an inclusion bias that likely led to underestimation of the true prevalence of PE, since a few patients may have had positive CUS performed directly at admission due to leg symptoms and would then have been treated without CTPA. Second, we could not provide a comprehensive radiation dose comparison between CTPA and perfusion scanning. Furthermore, we had no information regarding the affected side in patients with lower extremity symptoms. Heart rate data were not available for all patients, and some women did not undergo D-dimer testing. Finally, our low rate of PE (7%) may have prevented us from detecting significant differences between our two groups. However, our PE prevalence was nearly three times higher than previously described in pregnant women [37], and nearly double compared with data published by van der Pol et al [22]. Only Righini et al [23] reported a similar prevalence of PE (7.1%).

In conclusion, our present study did not identify clinical and laboratory parameters that were significantly associated with PE, emphasizing the important role of CTPA for excluding PE in pregnant women. Moreover, our findings indicated a low yield of PE-positive CTPA results, which suggest the need for new diagnostic strategies to safely exclude PE with fewer radiological examinations. However, over the last 17 years, CTPA was also able to reveal alternative diagnoses in one third of patients, while maintaining image quality and limiting radiation dose exposure.

## Abbreviations

CTDI_vol_: Volume CT dose indexes
CTPA: Computed tomography pulmonary angiography
CUS: Compression ultrasound
DVT: Deep vein thrombosis
MDCT: Multidetector computed tomography
PE: Pulmonary embolism
ROI: Region of interest
SD: Standard deviation
V/Q: Ventilation-perfusion lung scan
VTE: Venous thromboembolism
